# Pain and activity levels before and after platelet-rich plasma injection treatment of patellar tendinopathy: a prospective cohort study and the influence of previous treatments

**DOI:** 10.1007/s00264-012-1540-7

**Published:** 2012-04-27

**Authors:** Taco Gosens, Brenda L. Den Oudsten, Erik Fievez, Paula van ‘t Spijker, Alex Fievez

**Affiliations:** 1 Department of Orthopaedics and Traumatology, St Elisabeth Hospital Tilburg, Tilburg, The Netherlands; 2 Center of Research on Psychology in Somatic Diseases, Department of Medical Psychology, Tilburg University, Tilburg, The Netherlands; 3 Department of Education and Research, St. Elisabeth Hospital, Tilburg, Tilburg, The Netherlands; 4 Department of Orthopaedics, ORBIS Medical Center, Sittard, The Netherlands; 5 Department of Orthopaedics, Medinova Hospital, Rotterdam, The Netherlands

## Abstract

**Purpose:**

The aim of this study was to evaluate the outcome of patients with patellar tendinopathy treated with platelet-rich plasma injections (PRP). Additionally, this study examined whether certain characteristics, such as activity level or previous treatment affected the results.

**Methods:**

Patients (*n* = 36) were asked to fill in the Victorian Institute of Sports Assessment – Patellar questionnaire (VISA-P) and visual analogue scales (VAS), assessing pain in activities of daily life (ADL), during work and sports, before and after treatment with PRP. Of these patients, 14 had been treated before with cortisone, ethoxysclerol, and/or surgical treatment (group 1), while the remaining patients had not been treated before (group 2).

**Results:**

Overall, group 1 and group 2 improved significantly on the VAS scales (*p* < .0.05). However, group 2 also improved on VISA-P (*p* = .0.003), while group 1 showed less healing potential (*p* = 0.060). Although the difference between group 1 and group 2 at follow-up was not considered clinically meaningful, over time both groups showed a clinically significant improvement.

**Conclusion:**

After PRP treatment, patients with patellar tendinopathy showed a statistically significant improvement. In addition, these improvements can also be considered clinically meaningful. However, patients who were not treated before with ethoxysclerol, cortisone, and/or surgical treatment showed the improvement.

## Introduction

Like tendinopathy of the elbow, rotator cuff and Achilles tendon, tendinopathy of the patellar tendon (jumpers’ knee) is caused by degenerative tearing of collagen fibres rather than an acute inflammatory condition [1]. The underlying cause is considered to be a failed healing response due to poor tendon vascularity [2]. Lian et al. showed that hypovascularity plays a role in patellar tendinopathy, and also programmed cell death [3]. It causes substantial morbidity during sporting or working activities [4]. The pain and inability to perform sporting activities at the desired level warrants a search for adequate treatment. The range of treatments for patients with jumpers’ knee includes rest, ice, electrotherapy, massage, concentric and/or eccentric exercise, taping, corticosteroid injection, and surgery [5]. Feretti et al. found that conservative treatment did not produce sufficient relief of symptoms in those with jumper’s knee during a two-year follow-up. In addition, a substantial number of patients needed surgery, which did not always result in satisfactory outcomes [6]. Several studies have shown that extracorporeal shockwave therapy (ESWT) and sclerotherapy are promising [7, 8]. Recently, a higher level of evidence was reached showing ESWT to be ineffective in treating jumpers’ knee in in-season jumping athletes [9]. Since histopathological and biochemical evidence have indicated that the underlying pathology of tendinopathy is not an inflammatory tendinitis but a degenerative tendinosis, conservative therapy should be shifted from anti-inflammatory strategies (e.g., NSAID’s and steroids) to a more biological approach in which the tissue degeneration is arrested and even reversed. The use of platelet-rich plasma injections (PRP) has shown promising results in vitro [10] and in vivo, in lateral epicondylitis [11]. In contrast, the use of PRP in Achilles tendinopathy was judged not to be of value [12]. Therefore, the aim of this study was to evaluate the outcome of patients with patellar tendinopathy treated with PRP. Furthermore, this study examined whether certain characteristics, such as activity level and previous treatment affected the outcome.

## Methods

### Participants

This prospective cohort study measured pain and sporting ability in patients with chronic patellar tendinopathy (*N* = 36) before and after treatment with PRP. Of these patients, 14 had been treated with cortisone, ethoxysclerol and/or surgical treatment before PRP injection (group 1), while the other 22 patients had not received an injection or surgical treatment before (group 2). All patients had visited a physiotherapist and eccentric exercises had been performed exhaustively before being treated with PRP. The diagnosis of patellar tendinopathy was made on clinical grounds (pain on resisted extension of the knee located at the inferior pole of the patella). An additional MRI was performed in 21 (58.3 %) cases, showing oedema at the origin of the patellar tendon at the apex patellae in all cases. The mean follow-up period (in months) after being treated with PRP was 18.4 (SD = 4.9). The study was performed between March 2008 and June 2009 at the Medinova Hospital in Rotterdam (The Netherlands). The study protocol was approved by the Medical Ethics Committee.

### Platelet-rich plasma preparation

In the group randomised to receive PRP the patient’s own platelets were collected using the Recover System (Biomet Biologics, Warsaw, Indiana). This device uses a desktop-size centrifuge with disposable cylinders to isolate the platelet- and leukocyte-rich fraction from a small volume of the patient’s anticoagulated blood drawn at the time of the procedure. A 27-ml sample of whole blood was collected from the uninvolved arm into a 30-ml syringe that contained 3 ml of sodium citrate. The platelet-rich fraction was prepared according to the instructions for the use for the Recover System. Approximately 3 ml of PRP was obtained for each patient. The PRP was then buffered to physiological pH using 8.4 % sodium bicarbonate and Bupivacaine HCL 0.5 % with epinephrine (1:200000) was added. No activating agent was used. The total time from blood aspiration to injection in the patients was about 30 minutes. No specialised equipment, other than the centrifuge to process the Recover disposable, was required. All procedures were performed in the same office setting by an independent person certified for blood management.

### Injection technique

Approximately 1 ml of PRP with Bupivacaine HCL 0.5 % and epinephrine (1:200000) was injected directly into the area of maximum tenderness. Then the remaining PRP with Bupivacaine HCL 0.5 % with epinephrine (1:200000; ±4 ml) was injected by the investigator using a 22-g needle into the patellar tendon origin on the patella with a peppering technique. This technique involved a single skin portal and then five penetrations of the tendon.

### Post-procedure protocol

Immediately after the injection, the patient was rested in a supine position without moving the leg for 15 minutes. Patients were sent home with instructions to rest the leg for approximately 24 hours. If necessary, patients were allowed to use aminocetaphen, but the use of nonsteroidal anti-inflammatory medication was prohibited. After 24 hours, patients were given a standardised stretching protocol to follow for two weeks under the supervision of a physiotherapist. A formal eccentric muscle and tendon-strengthening program was initiated after this stretching. At four weeks after the procedure, patients were allowed to proceed with normal sporting or recreational activities as tolerated. The procedure for L-PRP preparation, injection technique and post-procedure protocol were identical to the procedure described for the lateral epicondylitis randomised controlled trial published previously, except for the standardised stretching protocol [11].

### Instruments

Patients were asked to fill in the Victorian Institute of Sports Assessment – Patellar questionnaire (VISA-P) and visual analogue scales (VAS) before and after treatment with PRP.

The VISA-P consists of eight questions [13, 14]. This instrument assesses (i) pain symptoms, (ii) function, and (iii) ability to play sport. As such, the VISA-P provides an impression of the severity of jumper’s knee. Six out of eight questions are scored on a visual analogue scale ranging from 0 (worst health) to 10 (perfect health). The maximum VISA-P score for an asymptomatic fully performing individual is 100 points and the theoretical minimum is 0 points. The internal consistency and test–retest reliability of the Dutch questionnaire was acceptable [14].

A VAS is an instrument to quantify the amount of pain reported by the patient. Scores can range from 0 (no pain) to 10 (severest pain). Patients were asked to fill in the level of pain during activities of daily life (ADL), work activities, and sport activities.

### Statistical analyses

Descriptive statistics (frequencies, percentages, means, and standard deviations) were used to present the available sociodemographic and clinical data at baseline. The groups (treated before or not treated before) were compared with Student t-tests or the Mann–Whitney U test and chi-square test. The paired-samples test was used to compare the VISA-P scores at follow-up with results assessed at baseline (when the total group was analysed). The Wilcoxon signed-ranks test was used to compare the VAS scores and VISA-P scores at follow-up with the results assessed at baseline (when group 1 and group 2 were analysed separately). In addition, effect sizes (i.e., an objective and standardised measure of the magnitude of an observed effect) were calculated. An effect size (ES) between 0.1 and 0.3 is small, 0.3–0.5 is moderate, and an ES greater than 0.5 is considered as large [15]. A 15-point difference on the VISA-P score was considered to represent a clinically meaningful difference [14]. The level of statistical significance was set at *P* < 0.05. All statistical analyses were performed using the Statistical Package for the Social Sciences (SPSS Chicago, IL, USA, version 17.0).

## Results

Table 1 shows the baseline characteristics of the total population of patients treated with PRP. The majority of the patients were male (63.9 %) and were not previously treated with cortisone, ethoxysclerol, and/or surgical treatment (*n* = 22; 61.1 %). Group 1 received different treatment combinations: one patient received cortisone, ethoxysclerol and surgical treatment (2.8 %), four patients received cortisone and ethoxysclerol (11.1 %), four patients received cortisone and surgical treatment (11.1 %), four patients received only cortisone (11.1 %), and one patient received ethoxysclerol (2.8 %). Table 2 shows the baseline characteristics of group 1 and group 2 separately. At baseline, group 1 and group 2 did not differ regarding demographic (age, sex) and clinical characteristics (duration of complaints in months, Tegner score) or the outcomes on VAS scores and VISA-P score.

**Table 1:** Baseline characteristics for the total population of patients with patellar tendinopathy treated with gravitational platelet separation

Characteristics	Values (*N* = 36)
Gender (men/women)	23 (63.9 %) / 13 (36.1 %)
Age (mean ± SD)	30.9 ± 12.6
Cortisone (yes/no)	13 (36.1 %) / 23 (63.9 %)
Ethoxysclerol (yes/no)	6 (16.7 %) / 30 (83.3 %)
Surgical treatment (yes/no)	5 (13.9 %) / 31 (86.1 %)
Duration of complaints in months (mean ± SD)	40.3 ± 28.4
VAS ADL score (mean ± SD)	4.0 ± 2.7
VAS work score (mean ± SD)	3.6 ± 3.9
VAS sport score (mean ± SD)	1.4 ± 0.8
VISA-P total score (mean ± SD)	57.7 ± 25.4

*SD* standard deviation,* VAS* visual analogue scale,* ADL* activities in daily life,* VISA-P* Victorian Institute of Sports Assessment – Patellar questionnaire

**Table 2:** Baseline characteristics for patients with patellar tendinopathy (group 1 and group 2)

Characteristics	Group 1 (*N* = 14)	Group 2 (*N* = 22)	*P*-value
Gender (men/women)	9 (64.3) / 5 (35.7)	14 (63.3) / 8 (36.4)	0.886
Age (mean ± SD)	28.7 ± 9.3	32.2 ± 14.8	0.399
Cortisone (yes/no)	13 (92.2) / 1 (7.1)	–	–
Ethoxy (yes/no)	6 (42.9) / 8 (57.1)	–	–
Surgical treatment (yes/no)	5 (35.7) / 9 (63.4)	–	–
Duration of complaints in months (mean ± SD)	38.5 ± 20.1	41.4 ± 33.0	0.774
Tegner score (VISA-P item 7)			
No, not at all	3 (21.4)	4 (18.2)	0.556*
Modified training or activity	6 (42.9)	8 (36.4)	
Full training/competition but not at same level as when symptoms began	5 (35.7)	6 (27.3)	
Competing at the same level as symptoms began	0 (0.0)	4 (18.2)	

*SD* standard deviation,* VISA-P* Victorian Institute of Sports Assessment – Patellar questionnaire

Percentages are given in parentheses. Group 1 is treated before with ethoxy, cortisone and/or surgical treatment, while group 2 is not

^a^ The Tegner score was dichotomised (0 = no not all/modified training or activity; 1 = full training/competing at the same level as symptoms began), since the expected cell count in the chi-square test based on the original scoring system was too low

### Pain and sport activities

Before treatment with PRP, only two patients (5.6 %) indicated that when they had no pain during sports, they were able to perform up to 40 minutes without any pain. After treatment with PRP, eight patients (22.2 %) were able to exercise without pain. These patients were able to play for at least 90 minutes without any pain, except for one patient who was able to play 40–60 minutes without pain. Before treatment, 13 patients (36.1 %) had pain, but were able to continue performing (score range: 60 minutes or more). After treatment, these numbers increased to 24 patients (66.7 %; score: 60 minutes or more). Initially, 11 patients (30.5 %) had such pain that they were not able to finish the training (score range: 30 minutes or more). However, after PRP treatment only four patients (11.1 %) had to quit during a training session (score range: 30 minutes or more).

### Changes in pain after PRP injection

The total group significantly improved after being treated with PRP. That is, mean scores on VISA-P improved from 40.1 (SD = 15.6) to 57.7 (SD = 25.4), with t(35) = −3.986, *p* < 0.0001, and ES = 0.56. VAS ADL decreased from 5.9 to 2.7 (*p* < 0.0001, ES = −0.51), VAS work decreased from 6.3 to 3.2 (*p* < 0.0001, ES = −0.50), and VAS sport decreased from 8.50 to 4.61 (*p* < 0.0001, ES = −0.55).

### The effect of pre-treatment on outcome

Mean scores on VISA-P significantly improved in group 2 from 39.1 (SD = 16.6) to 58.6 (SD = 25.4), with t(21) = −3.339, *p* < 0.003, and ES = 0.59. Group 1 did not show significant improvement on VISA-P [t(13) = −2.060, *p* = 0.060]. However, VAS ADL scores, VAS sport scores, and VAS work scores significantly improved in both groups (see Table 3). Effect sizes ranged from −0.32 to −0.56. Both groups showed a clinically meaningful improvement on VISA-P. The difference between group 1 and group 2 at follow-up was not considered clinically meaningful.

**Table 3:** Means and standard deviations of VISA-P and VAS scores at baseline and follow-up

Evaluation	Baseline	Follow-up	P-value	Effect size
Group 1 (*n* = 14)				
VISA-P	41.8 ± 14.3	56.3 ± 26.2	0.093	−0.32
VAS ADL	6.5 ± 2.3	2.8 ± 2.2	0.005	−0.53
VAS work	6.9 ± 2.5	3.1 ± 2.5	0.005	−0.53
VAS sport	8.6 ± 0.9	3.8 ± 2.9	0.003	−0.56
Group 2 (*n* = 22)				
VISA-P	39.1 ± 16.6	58.6 ± 25.4	0.003	−0.45
VAS ADL	5.6 ± 2.9	2.6 ± 2.7	0.001	−0.51
VAS work	6.0 ± 3.0	3.2 ± 2.9	0.001	−0.49
VAS sport	8.5 ± 1.1	5.1 ± 3.2	<0.0001	−0.55

*VISA-P* Victorian Institute of Sports Assessment – Patellar questionnaire, *VAS* visual analogue scales, *ADL* activities in daily life

Group 1 was treated before with ethoxy, cortisone and/or surgical treatment, while group 2 was not

## Discussion

PRP treatment results in statistically significant improvement in patients with chronic patellar tendinopathy compared with the pre-injections status. Furthermore, these improvements can be considered clinically meaningful. Those patients who were not treated before with ethoxysclerol, corticosteroid, and/or surgical treatment before entering this prospective study, showed the largest improvement and thus showed the largest healing potential.

The use of platelet-rich plasma as a treatment for chronic patellar tendinopathy reflects its use in other tendons and ligaments. There is increasing interest in sports medicine and orthopaedics in endogenous growth factors to relieve symptoms of degeneration of tendons and to counteract the failed healing response, which is characteristic of these injuries, to potentiate a faster return to sports and daily life activities. Despite this interest, and apparent widespread use, there is a lack of high-level evidence from randomised clinical trials assessing the efficacy of PRP in treating ligament and tendon injuries. Basic science and animal studies and small case series reports on PRP injections for ligament or tendon injuries, but few randomised controlled clinical trials have assessed the efficacy of PRP injections and none have demonstrated scientific evidence of efficacy. Scientific studies should be performed to assess clinical indications, efficacy, and safety of PRP, and this will require appropriately powered randomised controlled trials with adequate and validated clinical and functional outcome measures and sound statistical analysis. Other aspects of PRP use that need to be determined are (1) volume of injection/application, (2) most effective preparation, (3) buffering/activation, (4) injection technique (1 depot vs. multiple depots), (5) timing of injection to injury, (6) single application versus series of injections, and (7) the most effective rehabilitation protocol to use after PRP injection [16].

This small series is not randomised, although the data were prospectively collected, and therefore the positive effect of PRP series does not represent the highest level of evidence. On the other hand, it is the largest series of patients with a chronic patellar tendinopathy treated with PRP. Other reports [17–20] in literature describing the use of PRP in patellar tendinopathy describe a positive influence of PRP on the course of chronic patellar tendinopathy, although all studies used, for instance, a different brand or different concentrations. However, none of these studies recognises the effect the previous treatments may have had, as in our series. Only in the study of Filardo et al. it was mentioned that after failing of previous treatments, PRP still succeeded to diminish symptoms of patellar tendinopathy [17]. We saw improvement of symptoms in our study in those refractory cases when taking VAS for pain in daily life, sports and work into account, but VISA-P scores did not show improvement. Although an MRI was performed in 21 cases before the injection of L-PRP, showing oedema at the origin of the patellar tendon at the apex patellae, in only nine cases was a post-injection MRI made, showing in fact a reduction of oedema in seven cases. This small number of MRI scans does not allow us to make a statement on the relation between resolving symptoms and the MRI appearance.

There was a significant difference between those that had chronic tendinopathy without previous failing therapies and those with chronic tendinopathy of the same duration but with previously failing treatments. In this study we measured large effect sizes in VAS for pain in daily life, sports and work after the injection of PRP, thus a large change in pain when PRP is injected, which is independent of whether patients were treated before or treated for the first time. The effect size for the VISA-P was in a different range, indicating less effect of the injection of PRP following previously failed treatments such as injection of steroids, ethoxysclerol or surgery. It is important to realize is that the duration of complaints in months was not statistically different between these two groups: the previously treated patients did not have a longer history of symptoms. The well-known negative effects of repeated steroid injections were recently emphasised by Coombs et al. [21] The effects of surgery and ethoxysclerol were not described previously in relation to the healing potential of a tendon, but this series shows that previously treated patients have a smaller healing potential.

It was also noted that the pain with sports decreased significantly but most patients were still not pain free. This is illustrated by the improvement in VAS, but not by the improvement in VISA-P—the VISA-P scores the ability to perform specific sporting activities without pain whereas the VAS scores the level of pain during daily activities (ADL), work activities, and sport activities. Although the athletes were satisfied with this result it remains a point of interest. In search for complete recovery a second procedure was considered, but not attempted because of the experimental nature of this treatment. Repeated injections might be beneficial in patients who had suboptimal results after the initial injection, although no evidence for a beneficial effect of more than one injection exists. On theoretical grounds, by studying the inflammation cascade in tendon repair, a reinjection after three to four weeks seems logical since at this stage the cell proliferation and matrix deposition activity will have peaked and can be expected to subsequently decline. However, at this time no true indication of what the result of a second injection would be can be determined.

Comparing our results with operative treatment in literature, we believe that these results may justify the implementation of a PRP injection before considering surgical treatment of patellar tendinopathy. Surgical treatment has been recommended when nonoperative treatment fails. Open tenotomy and debridement proved to be a safe and effective technique with 59 % excellent and 35 % good results [22]. These percentages are higher than the results presented here, especially with respect to sporting activities, but since operative procedures in tendons in an athlete may be detrimental for a career, PRP may be a safe and less invasive way to create tendon healing.

Several other limitations should be acknowledged. The needling/fenestration of tendinopathic tissue has a positive effect itself on tendon healing. This study was not aiming to show the difference between dry needling and the injection of L-PRP, but showed a poorer healing potential in those who had been previously treated, irrespective of the duration of the symptoms that were classified as chronic in all cases. In this study we used the multiple-penetration-of-the-tendon-technique. The reason for choosing this technique is, in our opinion, not scientifically supported, but was made on an empirical basis (we obtained good results in lateral epicondylitis) [11] and to closely follow the instructions provided by the manufacturer of the GPS III system (Biomet).

In this study, we used the clinically minimal difference of the VISA-P to compare not only the treatment groups, but also to determine whether such a difference is present over time. However, no study has been performed to show whether this clinically meaningful difference could be used over time.

Another limitation of this study, apart from the fact that it is a non-randomised and non-controlled study, is that although this manuscript presents one of the largest samples of patients with patellar tendinopathy treated with PRP, it is too small to analyse the effect of the different treatment combinations on the outcome. We were not able to show the logically existing relation between multiple previous treatments and a less optimal outcome, although the results were not as good as those patients who had previous failed injections with steroids or sclerosants. Both groups improved in VAS for pain in daily life, sports and work, but VISA-P shows no significant improvement in those who had been treated before compared to those who only received PRP. Whether this proves the lesser ability of PRP to jump-start the healing mechanism, which was stopped by ethoxysclerol, steroid or surgery, or just proves the worsening effect of those treatment methods, remains to be seen. Yet, the results suggest that there may be a relationship between prior treatments and efficacy of PRP therapy for chronic patellar tendinopathy. This relationship needs further studies powered to detect such a difference.
